# Identification for the cortical 3-Hinges folding pattern based on cortical morphological and structural features

**DOI:** 10.3389/fnins.2023.1125666

**Published:** 2023-03-09

**Authors:** Chunhong Cao, Yongquan Li, Lele Zhang, Fang Hu, Xieping Gao

**Affiliations:** ^1^The MOE Key Laboratory of Intelligent Computing and Information Processing, Xiangtan University, Xiangtan, China; ^2^Key Laboratory of Medical Imaging and Artificial Intelligence of Hunan Province, Xiangnan University, Chenzhou, China; ^3^Hunan Provincial Key Laboratory of Intelligent Computing and Language Information Processing, Hunan Normal University, Changsha, China

**Keywords:** cortical 3-Hinges folding pattern, cortical morphology and structure, gender differences, deep learning, SE-Unet

## Abstract

The Cortical 3-Hinges Folding Pattern (i.e., 3-Hinges) is one of the brain's hallmarks, and it is of great reference for predicting human intelligence, diagnosing eurological diseases and understanding the brain functional structure differences among gender. Given the significant morphological variability among individuals, it is challenging to identify 3-Hinges, but current 3-Hinges researches are mainly based on the computationally expensive Gyral-net method. To address this challenge, this paper aims to develop a deep network model to realize the fast identification of 3-Hinges based on cortical morphological and structural features. The main work includes: (1) The morphological and structural features of the cerebral cortex are extracted to relieve the imbalance between the number of 3-Hinges and each brain image's voxels; (2) The feature vector is constructed with the K nearest neighbor algorithm from the extracted scattered features of the morphological and structural features to alleviate over-fitting in training; (3) The squeeze excitation module combined with the deep U-shaped network structure is used to learn the correlation of the channels among the feature vectors; (4) The functional structure roles that 3-Hinges plays between adolescent males and females are discussed in this work. The experimental results on both adolescent and adult MRI datasets show that the proposed model achieves better performance in terms of time consumption. Moreover, this paper reveals that cortical sulcus information plays a critical role in the procedure of identification, and the cortical thickness, cortical surface area, and volume characteristics can supplement valuable information for 3-Hinges identification to some extent. Furthermore, there are significant structural differences on 3-Hinges among adolescent gender.

## 1. Introduction

Cortical folding patterns quantify the human cerebral cortex, which is highly curled and folded into convex gyri and concave sulci during brain development. From these patterns, we can infer critical clues about cytoarchitecture (Van Essen, 1997; Fischl et al., 2008), neurodevelopment (Dubois et al., 2008), brain function and cognition (Thompson et al., 2004; Jiang et al., 2021). However, because the shapes of the gyri and the sulci are complex and variable across subjects, it is challenging to quantitatively analyze the cortical folding patterns, estimate precise cross-subject correspondences for them, and establish a mapping from them to brain function and cognition (Fischl et al., 2008). In particular, the location identification of the cortical folding has important clinical reference value for the prediction of human intelligence, the understanding of the brain functional structure (Jiang et al., 2018; Zhang et al., 2018a), and the diagnosis of neurological diseases (Huang et al., 2019).

Despite such difficulty, promising results have been achieved in solving these challenging problems. For example, learning from geological rock folding patterns analysis methods (Lisle, 1997; Li et al., 2010) defined the conjunction region of three gyral crests as a gyral hinge (denoted as 3-Hinges). Troubled by the formation mechanisms of 3-Hinges, Razavi et al. (2021) constructed a computational model of a growing brain and speculated that axonal wiring may be one of the most important contributors to 3-Hinges formation. The number, location, and shape of gyral hinges were used to quantitatively analyze the folding patterns of cerebral cortex (Nie et al., 2012; Ge et al., 2019; Huang et al., 2019). Gyral hinges receive an increasing attention not only because of their morphology, but also due to their importance in anatomy, axonal wiring diagram and brain functions: (1) they have thicker cortices (Li et al., 2010) and stronger axonal fiber connections (Ge et al., 2018); (2) they serve as the hubs of the cortico-cortical axonal fiber connective network (Zhang et al., 2020); and (3) they are more involved in global functional networks than other gyri (Zhang et al., 2020). According to recent studies, gyral hinges were suggested to serve as the anatomical landmarks, since corresponding gyral hinges across subjects were demonstrated to have unique and consistent structural connection patterns and brain function patterns (Zhang et al., 2020, 2022). In addition, some studies found that cortical folding pattern has significant differences among gender (Awate et al., 2010; Li et al., 2014). And these differences from the morphological structure of the cerebral cortex, especially the gyrus, may lead males and females to respond differently to the same cognitive activity (Charest et al., 2013; Hirjak et al., 2017).

Given the importance of gyral hinges, a more precise identification method is needed. In previous research, Yu et al. (2013) identified the gyral hinges by manual label. Chen et al. (2014) proposed a method based on energy minimization to identify the centroids of the gyral hinges with diffusion tensor imaging (DTI) derived fiber connectivity. Li et al. (2017) proposed an effective method for predicting the centroids of 3-Hinges based on DTI data using structural connection patterns and spatial distribution patterns. These methods significantly advanced the identification of 3-Hinges. However, they could not be easily generalized to the identification of 3-Hinges on large-scale cortical folding data since intensive manual intervention was involved. Subsequently, Chen et al. (2017) proposed a new representation of the cortical gyri pattern, named Gyral-net, which was automatically constructed as a gyral network. On this network, the nodes were automatically identified as gyral hinges, which are connected by gyral crests as edges (Chen et al., 2017; Zhang et al., 2018b). Despite the success of this automatic method, it takes a long time to only process the left or right brain of a single target at a time as the watershed algorithm and the tree marching algorithm are used such that it is hard to complete the identification task of gyral hinges on the dataset with a large amount of data.

Inspired by deep learning methods in many applications, Ge et al. (2019) applied convolutional neural network (CNN) to the cortical folding pattern recognition from functional magnetic resonance images (fMRI) to distinguish gyral hinges from other folding patterns. Although deep learning technique is promising in gyral hinge identification task due to its strength in latent feature exploration and utilization, the method in Ge et al. (2019) needs a precise cross-modality mapping to transfer the volumetric space of the fMRI data to the vertices on the cortical surface in T1-weighted MRI space, so did the method reported in Liu et al. (2022). Benefiting from the rich information of fMRI data, their work was influential on recognition of cortical folding pattern. However, instead of using the entire cortical fMRI data, they manually removed some data, according to cortical structure features. Furthermore, due to the huge variability of fMRI signals between individuals, both carried out their work at the individual level. In other words, a single model was trained for each subject, which consumed a lot of computing resources.

Therefore, this paper aims at developing a framework based on deep network models to realize the fast identification of cortical 3-Hinges simply from anatomic T1-weighted MRI and exploring whether there are structural differences on 3-Hinges among gender. The framework includes three major steps: Firstly, the morphological and structural features of the cerebral cortex are extracted from the reconstructed surface of the cerebral cortex. These features are then clustered into one feature vector per vertex using the K nearest neighbor algorithm. Secondly, based on this feature vector, cortical 3-Hinges folding regions are identified using a U-shaped neural network. Thirdly, the mean shift clustering algorithm is used to find the centroids of identified cortical 3-Hinges folding regions. Then, structural gender differences on 3-Hinges are discussed. The experimental results show that the proposed method can precisely recognize the locations of 3-Hinges and reveal the most contributive features to 3-Hinges identification, and there are significant differences in 3-Hinges morphological structure among adolescent gender.

## 2. Materials and methods

### 2.1. Overview

**Figure 1:**
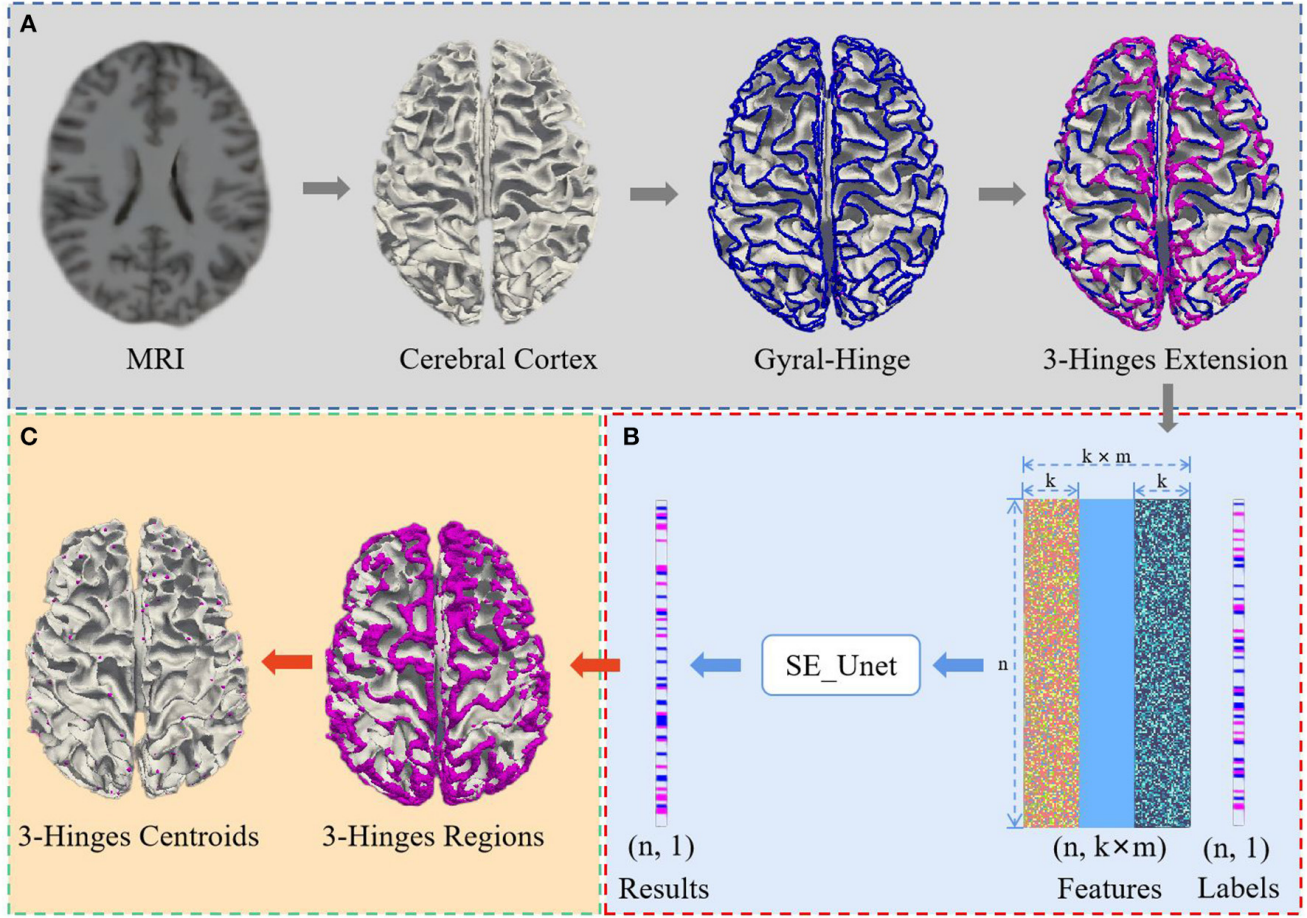
Overview of 3-Hinges locations identification framework. **(A)** Data preprocessing. **(B)** Identification of 3-Hinges regions. **(C)** Identification of 3-Hinges centroids.

We propose a 3-Hinges locations identification algorithm based on a deep network trained on the morphological and structural features of the cerebral cortex. As shown in Figure 1, the algorithm framework includes three main steps: data preprocessing, identification of 3-Hinges regions (n is the number of vertices, k is the result of the K nearest neighbor algorithm, and m is the number of fusion data) and identification of 3-Hinges centroids. These steps will be detailed in the following subsections.

### 2.2. Data preprocessing

#### 2.2.1. Feature extraction and preprocessing

**Figure 2:**
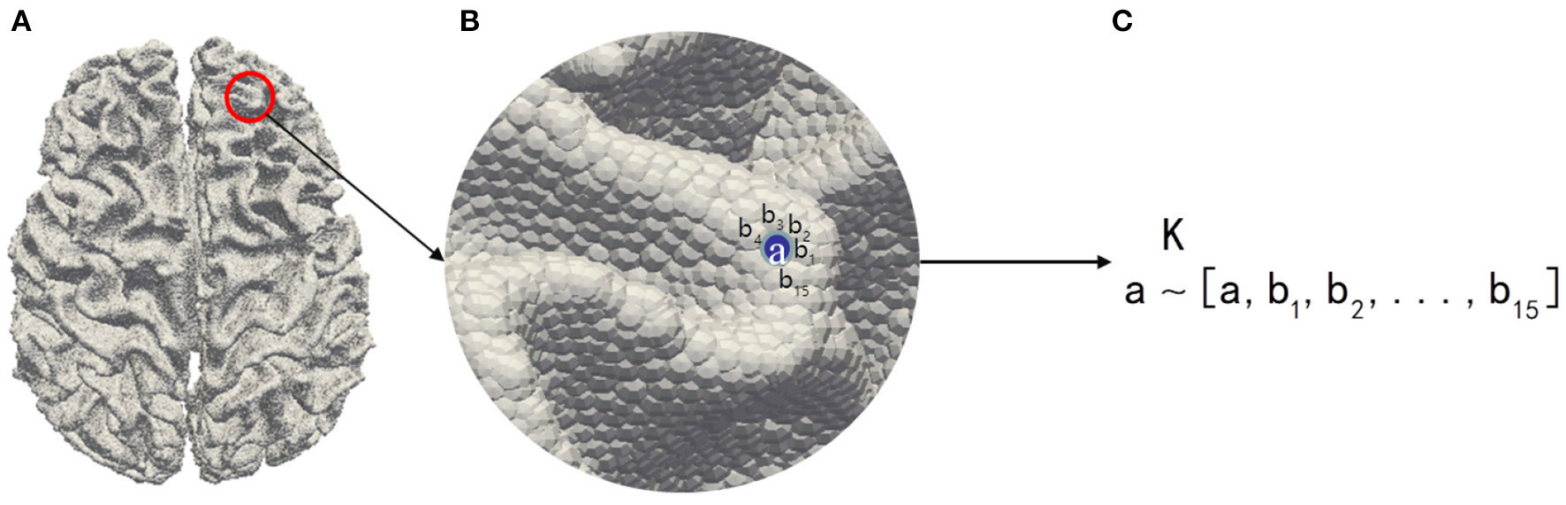
The illustration of feature preprocessing. **(A)** Cerebral cortex surface. **(B)** Zoom in view. **(C)** The feature vector [**a,b**_**1**_**,b**_**2**_**,...,b**_**15**_] of the vertex **a** is aggregated by the K nearest neighbor algorithm.

Considering the high ratio between the number of 3-Hinges centroids and the rest, we first use FreeSurfer (Fischl, 2012) for extracting features from the MRI reconstructed cortex to reduce the quantity ratio of non-3-Hinges to 3-Hinges. In this paper, we extract the morphological and structural features such as cortical thickness (thick), cortical surface area (area), cortical volume (vol), average curvature (curv) and sulcus (sulc) value to avoid using all the voxels in one brain as the input of the network model. In addition, because there are correlations among adjacent vertices on the cortex surface, we establish the spatial relationship between the scattered features with the K nearest neighbor algorithm (Cover and Hart, 1967; Pedregosa et al., 2011), and aggregate the morphological and structural features into a feature vector. For example, as shown in Figure 2, in our experiments, to each vertex a on the cortex surface, 15 vertices (b_1_,b_2_,...,b_15_) in the neighborhood of the vertex a are selected as the single input feature experimentally.

#### 2.2.2. 3-Hinges regions label

In order to further alleviate over-fitting in the training because of the imbalance between the number of 3-Hinges centroids and all the vertices of the cerebral cortex, three steps are involved in labeling 3-Hinges vertices.

**Figure 3:**
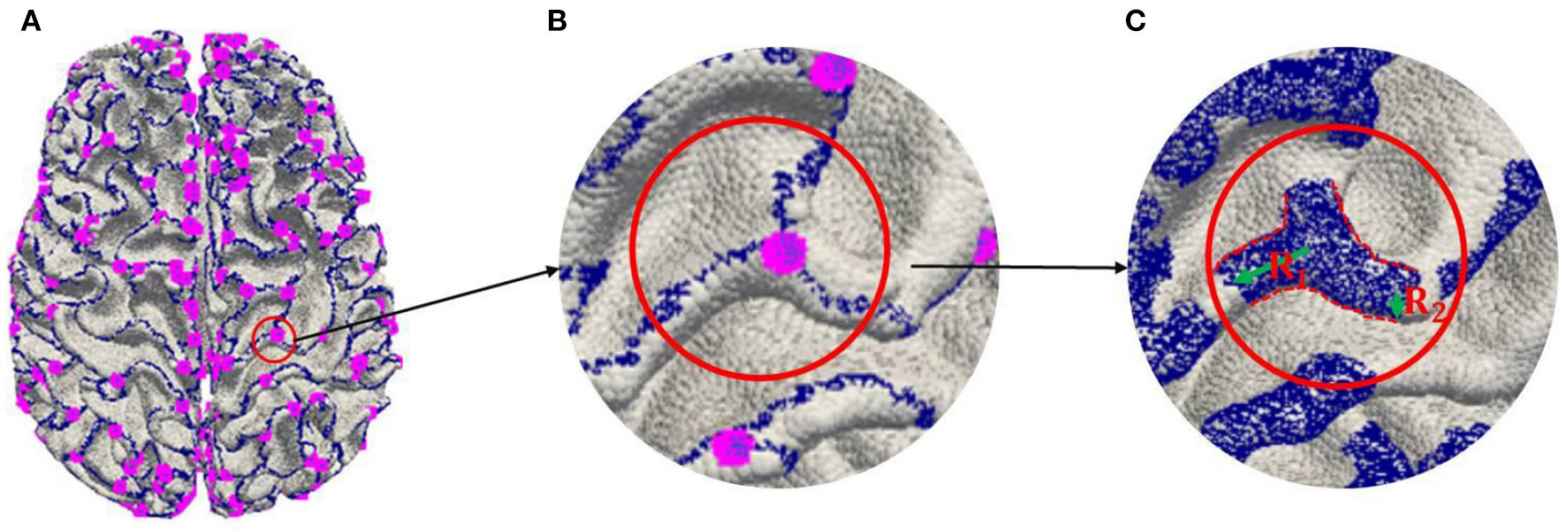
Labeling 3-Hinges regions. **(A)** Extracting 2-Hinges and 3-Hinges vertices by Gyral-net algorithm. **(B)** Zoom in 3-Hinges. **(C)** Expanding 2-Hinges and 3-Hinges regions.

(a) Extracting 2-Hinges and 3-Hinges vertices by the Gyral-net algorithm (the blue and the pink vertices are 2-Hinges and 3-Hinges vertices, respectively, as shown in Figure 3A. The readers can refer to Li et al. (2017) and Chen et al. (2017) about the detailed algorithm.
(b) Expanding 2-Hinges and 3-Hinges vertices into 3-Hinges region. As shown in Figure 3, we expand the vertices around the vertices generated by step (a). More specifically, two kinds of vertices are included in 3-Hinges region as shown in Figure 3C: i) cortex surface vertices in the spherical neighborhood within radius *R*_1_ (empirically set to 6 mm) of 3-Hinges vertices; ii) the cortex surface vertices in the spherical neighborhood within radius *R*_2_ (empirically set to 2 mm) of 2-Hinges vertices.
(c) Labeling 3-Hinges regions. We define the expanded region as 3-Hinges region shown as the blue region in Figure 3C. Each blue vertex is labeled as 1, and the rest is labeled as 0.

### 2.3. 3-Hinges regional identification

#### 2.3.1. Single feature SE-Unet network framework

**Figure 4:**
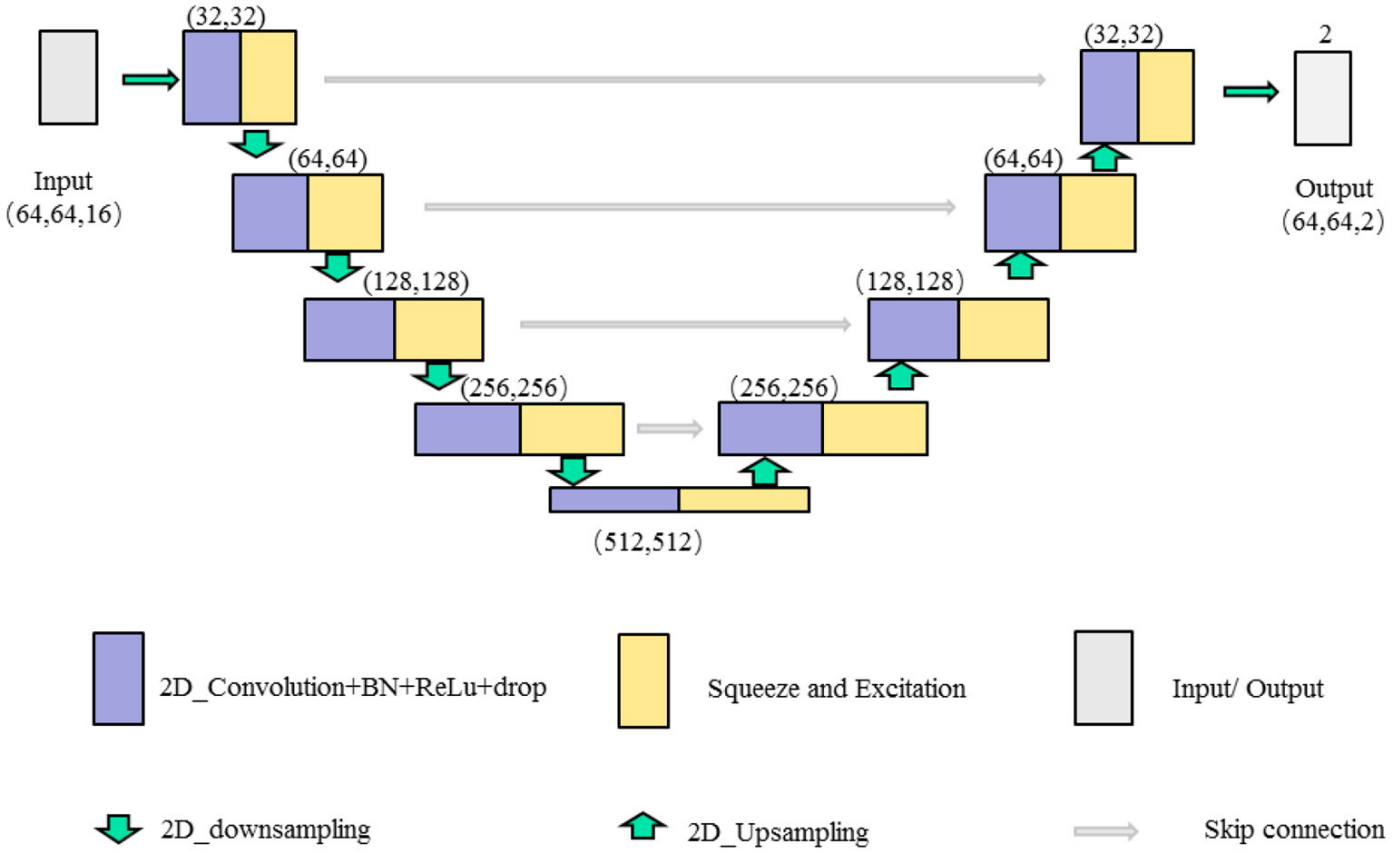
The illustration of single feature SE-Unet architecture.

**Figure 5:**
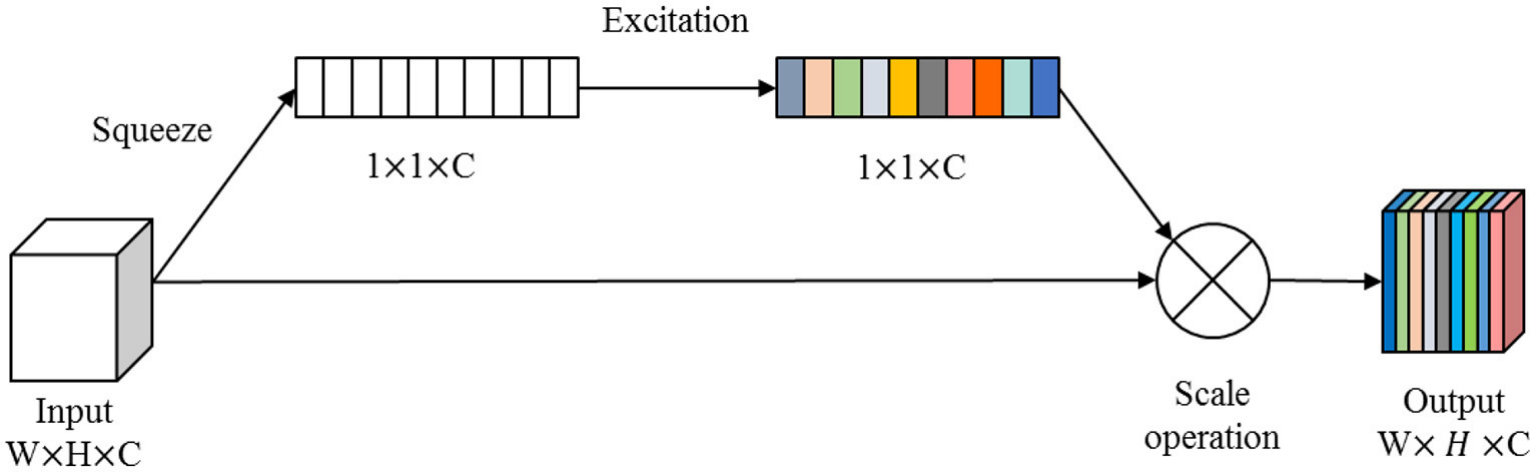
The architecture of the SE module.

In this paper, we combine the U-shaped network structure (Ronneberger et al., 2015) and SE (Squeeze and Excitation) module (Hu et al., 2020) to design a SE-Unet network framework for the morphological and structural features, which are used to identify 3-Hinges regions automatically. As shown in Figure 4, the network framework is a symmetrical U-shaped network with two paths, encoding (left side) and decoding (right side), and a total of 5 layers. The encoding paths consists of the repeated application of two 3 × 3 convolutions (purple block), a SE module (yellow block, the architecture is shown in Figure 5), and a 2 × 2 max pooling operation with stride 2 for down sampling (green down-arrow). At each down sampling step, we double the number of feature channels. The decoding paths consists of an up sampling of the feature map followed by a 2 × 2 convolution that halves the number of feature channels (green up-arrow), skip connections (gray right-arrow) concatenation with the corresponding feature map from the encoding path, two 3 × 3 convolutions and a SE module. Specifically, each convolution is followed by a layer of batch normalization (BN) and a layer of ReLu activation function. Meanwhile, a dropout layer is put between the convolutional layers to alleviate over-fitting. The input data is converted to the range of [0, 1] by maximum and minimum normalization before fed into the first module composed of two layers of convolutional blocks and the SE module. In addition, the softmax function is applied before the output of the SE-Unet network. In order to facilitate network training, the dimension of the network input is designed to be 64 × 64 × 16 in our experiments. Besides, to reduce the number of learning-parameters and time consumption, the 2D convolution is utilized in the proposed network.

#### 2.3.2. Multiple features fusion SE-Unet framework

**Figure 6:**
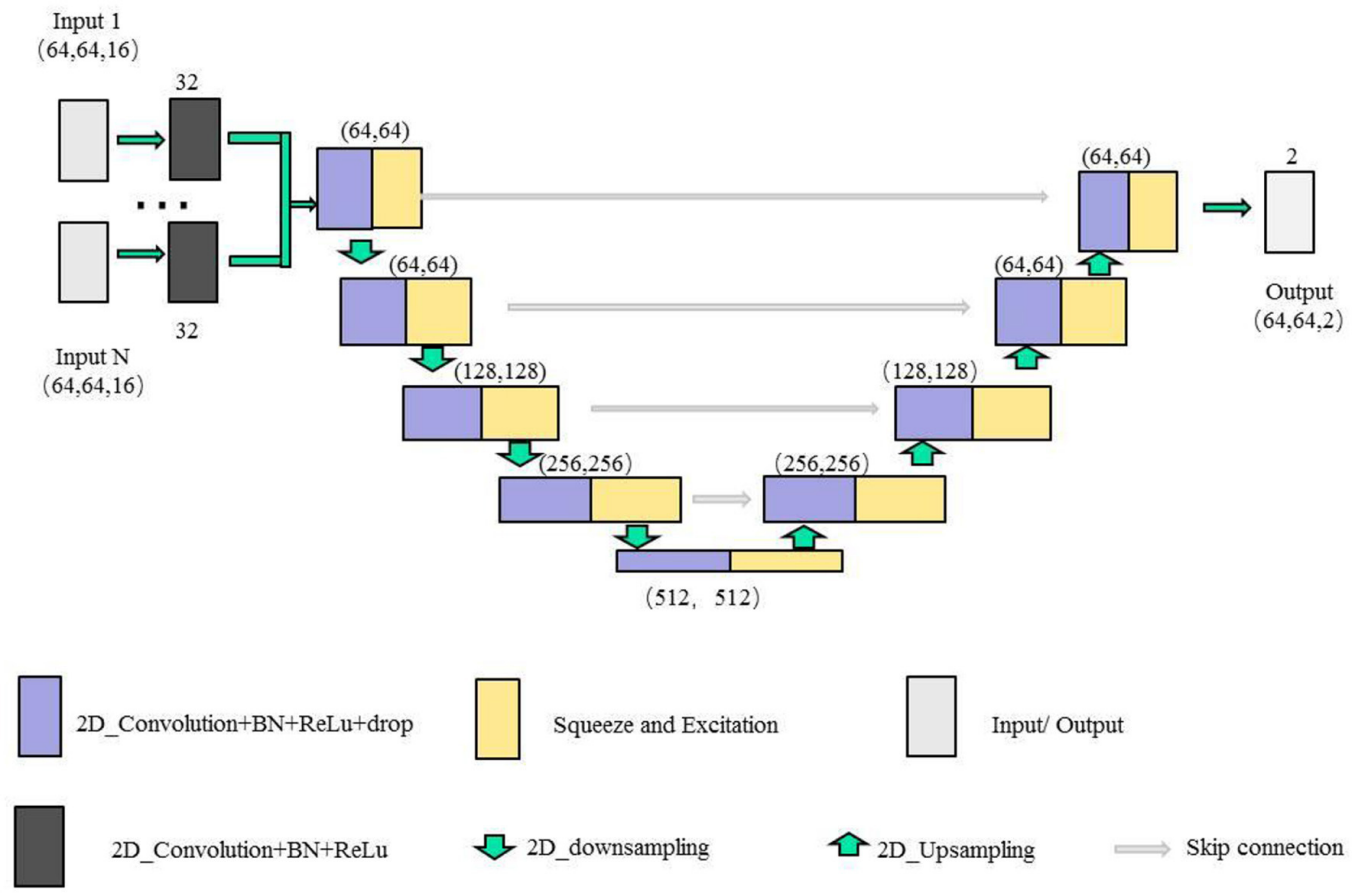
The illustration of the multiple features SE-Unet architecture.

For the extracted multiple feature vectors of the surface morphology and structure of the cerebral cortex, we design a multi-feature pre-fusion SE-Unet network framework to automatically extract 3-Hinges regions, as shown in Figure 6. The difference between this network structure and the single-feature SE-Unet network framework is that each feature in the input part of the network is first scaled by a convolutional block (including a 3 × 3 convolution layer, a layer of batch normalization (BN) and a layer of ReLu activation function), and then the scaled features are concatenated before being fed into the SE-Unet network.

### 2.4. 3-Hinges centroids identification

In order to identify the exact locations of 3-Hinges more precisely, we utilize the mean shift algorithm (Fukunaga and Hostetler, 1975; Comaniciu and Meer, 2002; Collins, 2003) to cluster the centroids of 3-Hinges regions. Considering that the algorithm does not need to pre-define the number of cluster centers and that the number of 3-Hinges centroids is also unknown in advance, the algorithm can directly determine the cluster centroids based on the calculated offset mean vector.

Assuming that a certain 3-Hinges region *X* in the left/right brain hemisphere is composed of the 3-dimensional coordinate vector *X*_*i*_(*i* = 1, 2, ..., *n*), i.e., *X* ∈ *R*^*n*×3^, the mean shift vector of *X*_*i*_(*i* ∈ {1, 2, ..., *n*}) in the original mean shift vector can be calculated by the formula (1):


(1) $$\begin{array}{l} M_{h}\left(X_{m}\right)=\frac{1}{K}\underset{X_{i}\in S_{h}}{\sum }\left(X_{i}-X_{m}\right), \end{array}$$

Where *S*_*h*_ is defined as the expression (Equation 2), *h* is the radius of 3-Hinges spherical region, and *K* is the number of coordinate vertices in 3-Hinges spherical region *X*.


(2) $$\begin{array}{l} S_{h}\left(X_{m}\right)=\left\{y:{\left(y-X_{m}\right)}^{T}\left(y-X_{m}\right)\leq h\right\}. \end{array}$$

However, the original mean shift algorithm assigns the same weight to each vertex in the region and regards them as the same importance. In fact, the closer the vertex is to the cluster center, the greater importance the vertex is to the cluster center. Therefore, the kernel function *G*(·) and weighted coefficients *w*(·) are introduced into the mean shift algorithm, and the formula (1) is modified as:


(3) $$M_{h}(X_{m})=\frac{\sum_{i=1}^{n}G_{H}(X_{i}-X_{m})w(X_{i})(X_{i}-X_{m})}{\sum_{i=1}^{n}G_{H}(X_{i}-X_{m})w(X_{i})},$$

Where *w*(*X*_*i*_) ≥ 0 is the weight corresponding to the coordinate vertex *X*_*i*_ according to the distance between *X*_*i*_ and *X*_*m*_. *G*_*H*_(*X*_*i*_−*X*_*m*_) is obtained by the expression:


(4) $$\begin{array}{l} \;G_{H}(X_{i}-X_{m})=\text{|}H{\text{|}}^{\frac{1}{2}}G_{H}(\text{|}H{\text{|}}^{\frac{1}{2}}(X_{i}-X_{m})),\\ with\;G_{H}(x)=-(\frac{1}{\sqrt{2\pi s}}e^{-\frac{x^{2}}{2s^{2}}}{)}^{\prime },s\in constant, \end{array}$$

and *H* is a *d*×*d* bandwidth matrix, which can be the diagonal matrix $H=diag\left[h_{1}^{2},...,h_{d}^{2}\right]$ or the proportional unit matrix *H* = *h*^2^*I*. Considering that the later one only has one hyper-parameter *h*, we choose *H* = *h*^2^*I* in the mean shift algorithm to facilitate the identification of 3-Hinges. After simplification, our final mean shift vector can be expressed as Equation (5):


(5) $$M_{h}(X_{m})=\frac{\sum_{i=1}^{n}G_{H}(\frac{X_{i}-X_{m}}{h})w(X_{i})(X_{i}-X_{m})}{\sum_{i=1}^{n}G_{H}(\frac{X_{i}-X_{m}}{h})w(X_{i})},$$

then, the 3-Hinges centroid is updated as *X*_*m*_ = *X*_*m*_ + *M*_*h*_(*X*_*m*_).

## 3. Experimental results

In this section, we will introduce the data set, evaluation metrics, and network parameters. At the same time, we analyze the single feature and multiple combined features that are most relative to 3-Hinges. We also verify the generalization of the method on the adult data set. The code is available at https://github.com/GuardianTree/code.

### 3.1. Training

We evaluated our method on T1-weighted MR images from adolescent and adult data sets.

#### 3.1.1. Data sets

*The Adolescent MRI Data*: In this study, the MRI from the Adolescent Brain Cognitive Development (ABCD) NIMH Data Archive (NDA) Study is used where all the subjects are between 9 and 10. Compared with infant brains, the brain at this age is considered to be relatively, with discriminative cortical folding patterns. The ABCD data set has been processed in accordance with the MRI preprocessing procedure mentioned by Jenkinson et al. (2002), Pfefferbaum et al. (2018), and Hagler et al. (2019). Limited by computational resources, we randomly select 1,000 brain MRI data from ABCD NDA Release 1.1. It is noted that the proposed method can be applied to many datasets including the above-mentioned datasets.

For the ABCD data set, there are approximately 330,000 vertices on the surface of the cerebral cortex of each sample. In order to facilitate the use of deep learning method, we will unify the features extracted from each sample to 331,776 (=64*64*81), that is, we add the morphological and structural features of the vertices that do not meet the requirements to 331,776 with a value of 0. After sampling and shape transformation, the features of each subject are divided into 81 blocks of size (64, 64, 16). Therefore, there are 72,900 blocks in the training set and 8,100 blocks in the test set.

*The Adult MRI data*: In this experiment, the adult data set is the 1,200 data set released by the Human Connectome Project (HCP). The HCP data set contains images of a total of 1,200 normal young people aged 22–35. The detailed process of HCP data set parameters can be found in the processing of Van Essen et al. (2013). In order to verify the generalization of the adult data, 110 adults were selected from the HCP data set (http://www.humanconnectomeproject.org/data/).

For the HCP data set, there are approximately 360,000 vertices on the surface of the cerebral cortex of each subject. After the same processing as the ABCD data set, the extracted features are divided into 90 blocks, and the final HCP data set has 9,900 blocks with the size of (64, 64, 16).

### 3.2. Evaluation metrics

In this paper, three metrics are used to evaluate 3-Hinges regions identification performance in the experiment, i.e., Precision, Recall, and F1. In addition, in the process of identifying the locations of 3-Hinges centroids, the prediction error (PreE) calculated by the Euclidean distance between the predicted centroids and the labels is used as the evaluation metric. Lh-PreE, rh-PreE and mean-PreE represent the average values of the prediction error of 3-Hinges centroids on the left, right, and whole brain, respectively. The smaller the value of the PreE is, the closer the predicted 3-Hinges centroids locations are to the true 3-Hinges centroids locations.

### 3.3. Network parameters

In this experiment, we implement the SE Unet Network with the Keras framework, where the RMSprop optimizer Wilson et al. (2017) and Hinton et al. (2012) is used for optimization training. The initial learning rate is set as 0.05, which is decayed exponentially after each epoch. The batch size is set as 40, the epoch is set as 150, the convolution kernel size is set as 3 × 3, and the momentum parameter in the batch normalization layer is set as 0.6. The activation function layer is the ReLu function, the drop layer parameter is set as 0.2, the parameters in down-sampling and up-sampling are both set as 2 × 2. In order to obtain the true objective maximization of 3-Hinges regions, the Dice loss is selected as the training loss function.

### 3.4. 3-Hinges identification

#### 3.4.1. Single feature result analysis

In the experiment, we first give the results of identifying 3-Hinges regions using the baseline U-net, and list the results using the proposed SE-Unet under different dimensionality reduction coefficients (r), which is a hyper-parameter in the SE module.

Then, based on the recognition of 3-Hinges regions, the mean shift clustering algorithm is used to identify the centroids of 3-Hinges regions. As shown in Table 1, when the hyper-parameter r is set to 24, the F1 score reaches 60.78, and the mean-pre of the predicted 3-Hinges centroids on the entire brain of all test set individuals is 5.56. Meanwhile, in the same experimental environment, the time consumption of our algorithm is about 4 min, which is far less than the Gyral-net method, indicating that our algorithm can identify the locations of 3-Hinges centroids more quickly.

**Table 1:** The identification results of different methods.

**Data**	**Methods**		**3-Hinges regions (%)**			**3-Hinges centroids (mm)**			**Time** ** (min)**
			**Precision**	**Recall**	**F1**	**lh-** **PreE**	**rh-** **PreE**	**mean-** **PreE**	
MRI	Gyral-net		-	-	-	-	-	-	82.30
sulc	Unet+ mean shift		56.23	65.71	60.56	5.58	5.63	5.60	4.06
sulc	SE_Unet+ mean shift	*r* = 8	55.32	67.28	60.67	5.55	5.63	5.59	4.05
		*r* = 16	55.47	67.12	60.70	5.56	5.61	5.58	4.07
		*r* = 24	55.74	66.93	60.78	5.52	5.60	5.56	4.06
		*r* = 32	56.49	64.35	60.12	5.54	5.59	5.56	4.05

**Figure 7:**
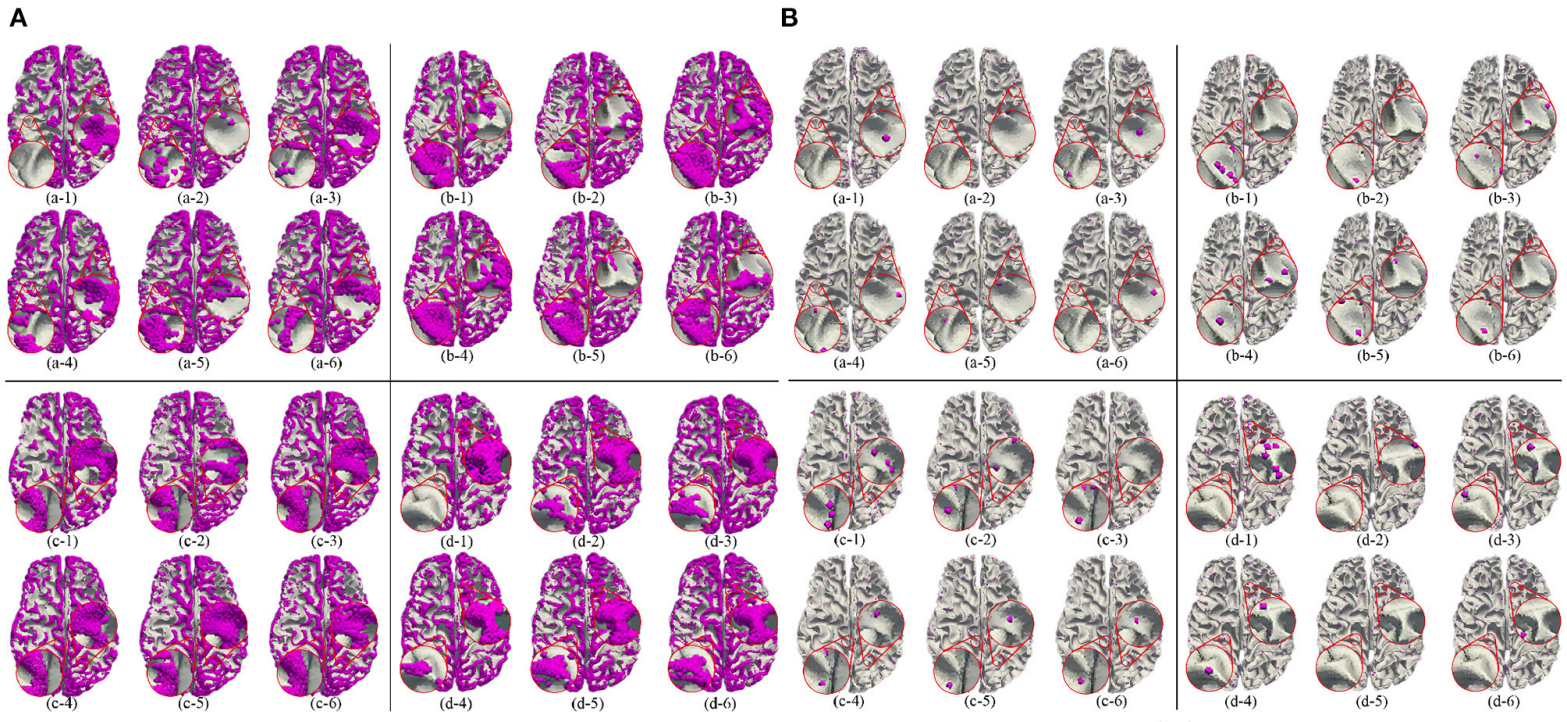
3-Hinges regions **(A)**/centroids **(B)** visualized results using different features. Letters (a–d) represent different individuals. Numbers (1–6) indicate label, area, curv, sulc, thickness, and volume, respectively.

Besides, we report the precision, recall and F1 under the other morphological and structural features of the cerebral cortex, such as cortical thickness, surface area, volume, average curvature and sulcus value, as shown in Table 2. We can see that under the same conditions, the sulc recognition results outperform those of other features. In addition, in the same experimental environment, compared with the Gyral-net method, the time required for our method is about 4 min, which are far less than Gyral-net method. As shown in Figure 7, we can observe that 3-Hinges regions identified by the sulcus value feature contains more 3-Hinges vertices which are close to the real 3-Hinges centroids. In some subjects, our predicted results are even more accurate than the labels annotated by Gyral-net such as those in (d-1) and (d-4) of Figure 7B.

**Table 2:** The identification results on different single features.

**Data**	**Methods**	**3-Hinges regions (%)**			**3-Hinges centroids (mm)**			**Time** ** (min)**
		**Precision**	**Recall**	**F1**	**lh-** **PreE**	**rh-** **PreE**	**mean-** **PreE**	
MRI	Gyral-net	-	-	-	-	-	-	82.30
sulc	SE-Unet+ mean shift	55.74	66.93	60.78	5.52	5.60	5.56	4.06
curv		46.36	55.38	50.42	7.68	7.65	7.66	4.06
vol		47.84	55.21	51.23	6.92	6.86	6.89	4.09
area		44.60	43.49	44.01	8.46	8.46	8.45	4.07
thick		46.72	54.98	50.48	7.40	7.48	7.44	4.01

#### 3.4.2. Multiple features result analysis

Based on the experiment results of the single feature above, we try to improve 3-Hinges locations identification by fusing different features. In this section, we choose to use feature fusion in the early stage to explore the impact of fusion features on 3-Hinges locations identification, as shown in Table 3.

**Table 3:** The identification results of multi-features fusion.

**Data**	**Methods**	**3-Hinges regions (%)**			**3-Hinges centroids (mm)**			**Time** ** (min)**
		**Precision**	**Recall**	**F1**	**lh-** **PreE**	**rh-** **PreE**	**mean-** **PreE**	
MRI	Gyral-net	-	-	-	-	-	-	82.30
sulc+vol	SE-Unet +mean shift	55.91	70.33	62.21	5.26	5.25	5.25	4.08
sulc+thick		56.43	70.29	62.54	5.22	5.24	5.23	4.09
sulc+curv		50.25	74.87	59.84	5.94	5.93	5.94	4.07
sulc+area		56.13	69.30	61.91	5.52	5.51	5.51	4.09
vol+thick		49.21	56.80	52.68	6.82	6.84	6.83	4.11
vol+curv		43.97	42.70	41.80	7.37	7.33	7.35	4.15
vol+area		47.10	61.65	53.29	6.89	6.90	6.90	4.08
thick+curv		39.38	54.72	44.48	8.13	8.10	8.11	4.10
thick+area		47.71	55.78	51.30	7.49	7.47	7.48	4.13
curv+area		15.94	49.25	24.00	9.25	9.25	9.25	4.11
sulc+thick+vol		56.91	69.72	62.58	5.16	5.20	5.18	4.12
sulc+thick+area		56.59	69.23	62.15	5.21	5.22	5.21	4.11
sulc+vol+area		56.45	69.72	62.29	5.16	5.21	5.18	4.13
sulc+thick+vol+area		57.02	69.53	62.54	5.15	5.16	5.15	4.17
sulc+thick+vol+area+curv		51.70	74.02	60.35	5.55	5.65	5.60	4.32

The result under the fusion of sulc+thick in 3-Hinges regions reaches 62.54, and the mean-PreE is only 5.23 mm. With sulc+curv the results are worse than that of a single sulc feature, which shows that the curv feature inhibits the sulc feature from identifying 3-Hinges locations. Similar conclusions are obtained from other feature combinations. We also get the optimal results with 3–5 features where it can be seen that more features do not improve the recognition results significantly, although the combination of sulc+thick+vol+area achieves better results at the cost of more time consumption. Some visualized results predicted by fusion of sulc feature and other structural features are shown in Supplementary Figures S1–S4. In general, our proposed method can predict some 3-Hinges points that are not labeled, such as a larger version of the left brain of individual a and d, and the right brain of individual b. Moreover, there are less 3-Hinges points, which are more likely to be representative in the same 3-Hinges region by using mean shift.

#### 3.4.3. Correlation analysis with gender

We performed a correlation analysis between 3-Hinges cortical structural features classification accuracy and the subjects' gender, as shown in Table 4. In 100 test subjects, there are 51 females and 49 males. In single cortical structural feature tasks, there is not a significant correlation between 3-Hinges classification accuracy of one cortical structural feature and gender. But compared with the others, the result of cortical structural feature of the sulc have a closer association with gender (*r* = 0.18, *p* = 0.07). In two cortical structural features tasks, there is a significant correlation between 3-Hinges classification accuracy and gender (*r* = 0.21, *p* = 0.03 and *r* = 0.22, *p* = 0.03 for curv_sulc and sulc_thickness, respectively). In both three and four cortical structural features tasks, there are also significant correlations between 3-Hinges classification accuracies and gender (*r* = 0.24, *p* = 0.02 and *r* = 0.22, *p* = 0.03 for area_sulc_volume and area_curv_sulc_thickness, respectively). It is worth noting that in five cortical structural features tasks, there is the most significant correlation between 3-Hinges classification accuracy and gender (*r* = 0.25, *p* = 0.01). With the increasement of multiple cortical structural features, the correlation between 3-Hinges classification accuracy and gender becomes more and more significant, either the Pearson correlation coefficient or the *p*-value. Furthermore, all of the correlations are positive. It indicates that 3-Hinges structure of adolescent males is significantly different to that of females. Compared with other cortical folding regions, 3-Hinges regions are more prominent in males. This reslut is consistent with the previous study on gender differences in cerebral cortical folding patterns, in which the fraction of the cortical surface that was convex (predominantly gyri including 3-Hinges) was significantly higher in males (Awate et al., 2009). In other words, structural roles that 3-Hinges within adolescent males and females plays do change remarkably.

**Table 4:** The correlation analysis between 3-Hinges regions identification accuracy and the gender in adolescents.

**Data**	***r***	***p*-value**	**Data**	***r***	***p*-value**
sulc	0.18	0.07	sulc+curv+vol	0.20	0.05*
curv	0.10	0.33	sulc+vol+area	0.24	0.02^*^
vol	0.11	0.27	sulc+thick+area	0.21	0.03^*^
area	0.16	0.10	sulc+thick+curv	0.21	0.04^*^
thick	0.15	0.14	sulc+thick+vol	0.19	0.06
sulc+area	0.19	0.06	sulc+vol+area+curv	0.21	0.04^*^
sulc+curv	0.21	0.03^*^	sulc+thick+area+curv	0.22	0.03^*^
sulc+vol	0.22	0.03^*^	sulc+thick+vol+area	0.18	0.08
sulc+thick	0.22	0.03^*^	sulc+thick+vol+curv	0.21	0.03^*^
sulc+area+curv	0.22	0.03^*^	sulc+thick+vol+area+curv	0.25	0.01^**^

The females and the males are labeled as 0 and 1, respectively.

* represents *p*-value < 0.05, which means general significant correlation;

** represents *p*-value < 0.01, which means extremely significant correlation.

### 3.5. Generalization

**Figure 8:**
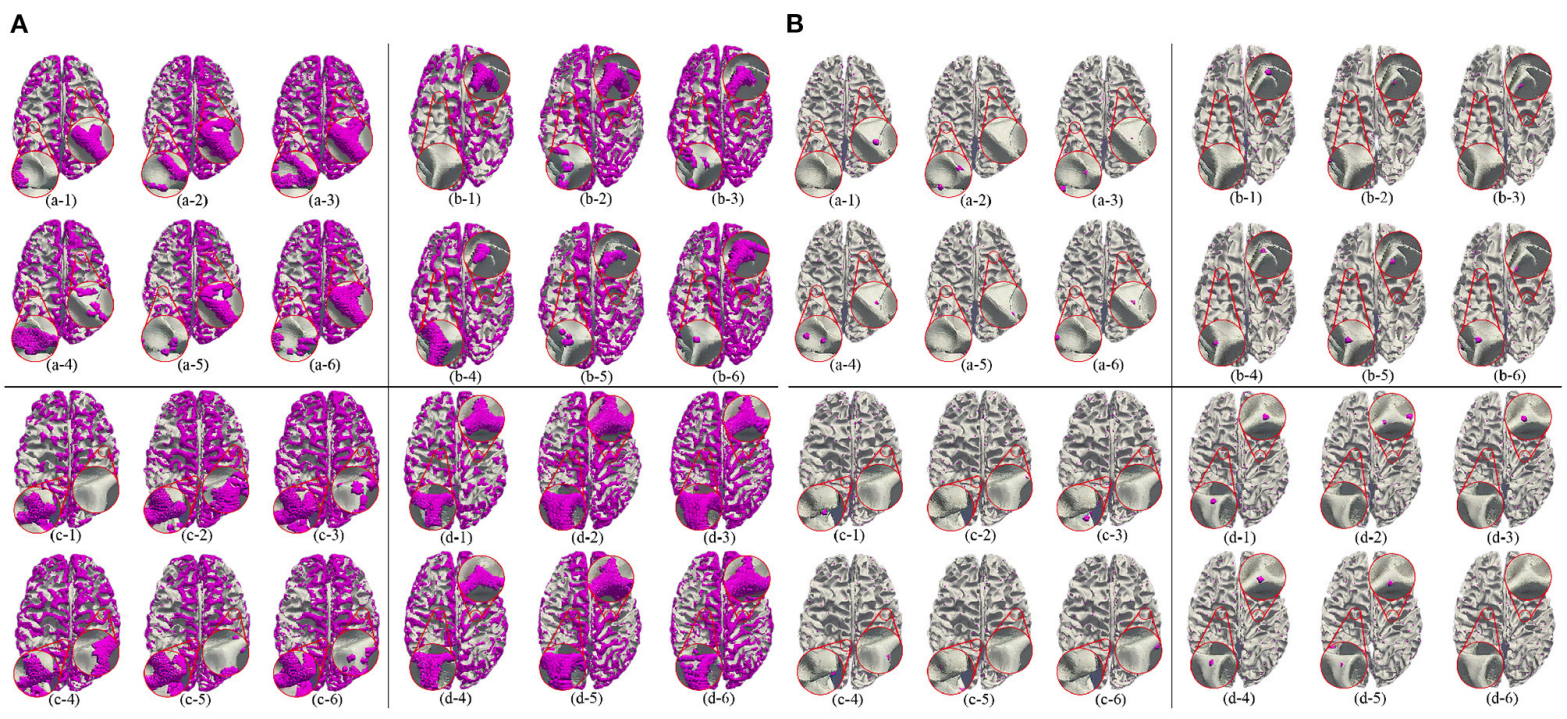
3-Hinges regions **(A)**/centroids **(B)** visualized results using different features on HCP. Letters (a–d) represent different individuals. Numbers (1–6) indicate label, area, curv, sulc, thickness, and volume, respectively.

In this section, we test the adult data directly using the model trained on the ABCD data set. The results are shown in Table 5. It shows that we can get the consistent conclusions as the ABCD data set, although the accuracy is less than that of the ABCD data set. By analyzing and comparing the identified 3-Hinges regions and centroids, we find that on one hand, the adult brain is more mature than the adolescent brain, and its cerebral cortex folding is more complicated, which increases the difficulty of 3-Hinges' identification. On the other hand, the number of the vertices contained in the identified 3-Hinges regions is reduced, which results in less 3-Hinges centroids. However, as shown in Figure 8, the proposed method can still identify 3-Hinges points in some cases that are not correctly labeled by Gyral-net.

**Table 5:** The identification results of HCP data set.

**Data**	**Methods**	**3-Hinges regions (%)**			**3-Hinges centroids (mm)**			**Time** ** (min)**
		**Precision**	**Recall**	**F1**	**lh-** **PreE**	**rh-** **PreE**	**mean-** **PreE**	
MRI	Gyral-net	-	-	-	-	-	-	82.30
sulc	SE-Unet +mean shift	50.98	56.94	53.67	6.54	6.50	6.52	4.14
curv		46.16	59.01	51.65	8.13	8.25	8.19	4.18
vol		41.19	54.63	46.91	7.86	7.77	7.81	4.17
area		42.02	45.82	43.79	9.17	9.19	9.18	4.16
thick		42.16	49.50	45.48	8.09	8.22	8.16	4.13
sulc+thick		50.23	46.90	48.26	6.19	6.18	6.18	4.18
sulc+thick+vol		51.19	45.02	47.55	6.09	6.11	6.10	4.24
sulc+thick+vol+area		52.49	40.26	45.14	6.05	6.06	6.05	4.27
sulc+thick+vol+area+curv		47.76	56.18	50.73	6.52	6.47	6.49	4.32

## 4. Discussion and conclusion

In this article, we propose a SE-Unet algorithm to identify 3-Hinges regions based on the extracted brain morphological features. The algorithm first extracts the morphological and structural features of the brain, then utilizes the K nearest neighbor algorithm to establish the spatial index relationship between the scattered features and aggregates the extracted neighborhood features into a feature vector to improve the performance of the algorithm. At the same time, the deep U-shaped network structure and the squeeze excitation module are merged to learn the correlation of the channels in the feature vector, resulting in the automatic weight assignment of useful cortical structure feature channels. The cortical 3-Hinges regions can therefore be quickly identified. In addition, The mean shift algorithm is used to identify the centroids of the cortical 3-Hinges, considering that the cortical 3-Hinges is similar or identical in shape, which results in the inaccurate reflection of the cortical folding patterns. Through the comparative analysis of the experimental results of using a single feature and multiple features, we can conclude that the single sulc feature is sufficient to identify 3-Hinges. Meanwhile, the fusion of sulc, thickness, volume and area features can well identify 3-Hinges at the price of more time consumption. In consideration of the performance difference of identifying 3-Hinges between adolescent males and females, it is obvious that there are significant structural differences between males and females. In addition, we also carried out generalization verification on the adult dataset. Although our method improves the current Gyral-net to some extent, there are still room for improvement. We will aim for high accuracy prediction of the cortical 3-Hinges from both structural MRI and functional MRI.

## Data availability statement

The raw data supporting the conclusions of this article will be made available by the authors, without undue reservation.

## Ethics statement

The HCP study was ethically approved by the Washington University Institutional Review Board (IRB). Written informed consent from the participants' legal guardian/next of kin was not required to participate in this study in accordance with the national legislation and the institutional requirements. Written informed consent was not obtained from the individual(s), nor the minor(s)' legal guardian/next of kin, for the publication of any potentially identifiable images or data included in this article.

## Author contributions

CC: supervision, writing–review, editing, validation, and project administration. LZ: methodology, writing–original draft, and coding. YL: writing–original draft, formal analysis, visualization, and coding. FH: supervision and project administration. XG: supervision and writing–review. All authors contributed to the article and approved the submitted version.
