# Supplementary material for: Identification for the cortical 3-Hinges folding pattern based on cortical morphological and structural features
Source: Front Neurosci. 2023 Mar 9;17:1125666. doi: 10.3389/fnins.2023.1125666 (PMC10034048; doi:10.3389/fnins.2023.1125666)
Supplement: Supplementary file 1 [file Data_Sheet_1.docx]

**Supplemental Materials**


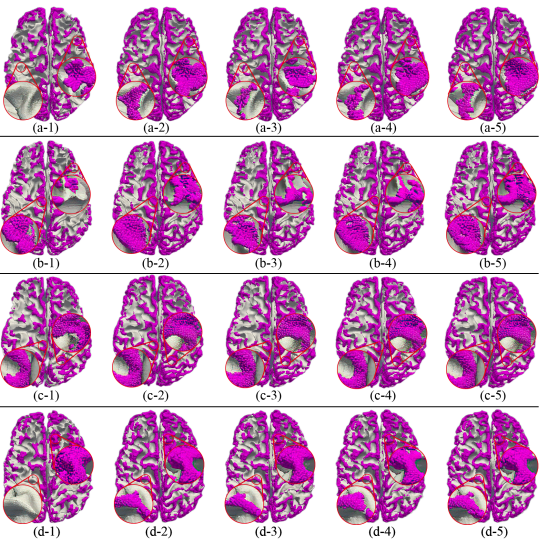

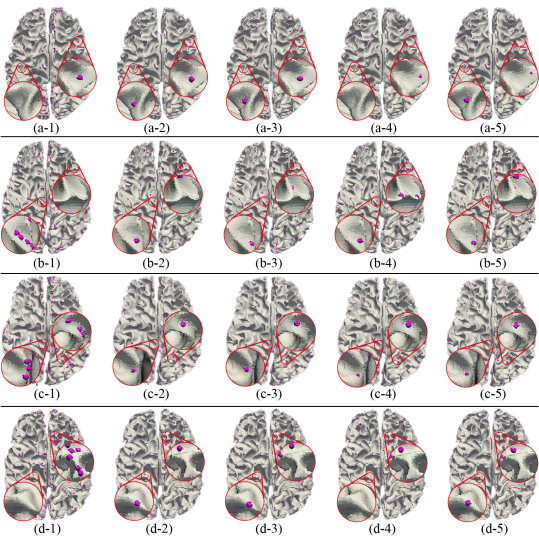


**(A) (B)**

Figure S1: The 3-Hinges regions (A)/centroids (B) visualized results using two features. Letters (a-d) represent different individuals. Numbers (1-5) indicate label, area+sulc, curv+sulc, sulc+thickness, and sulc+volume, respectively.


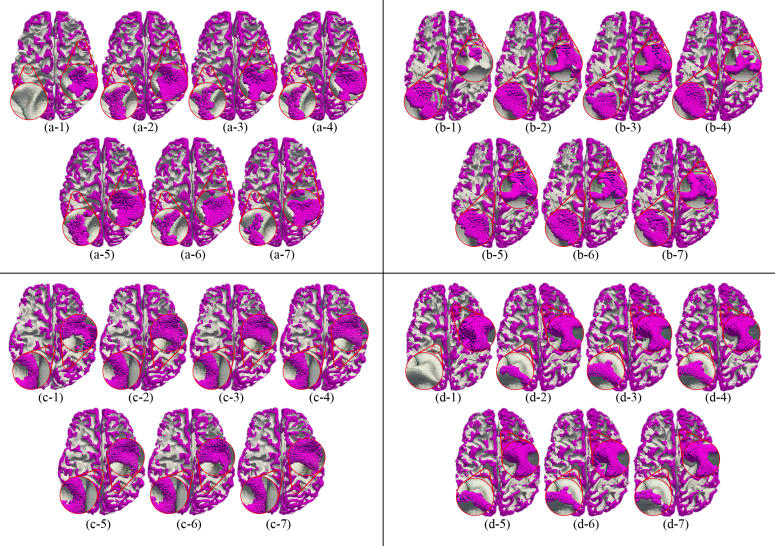

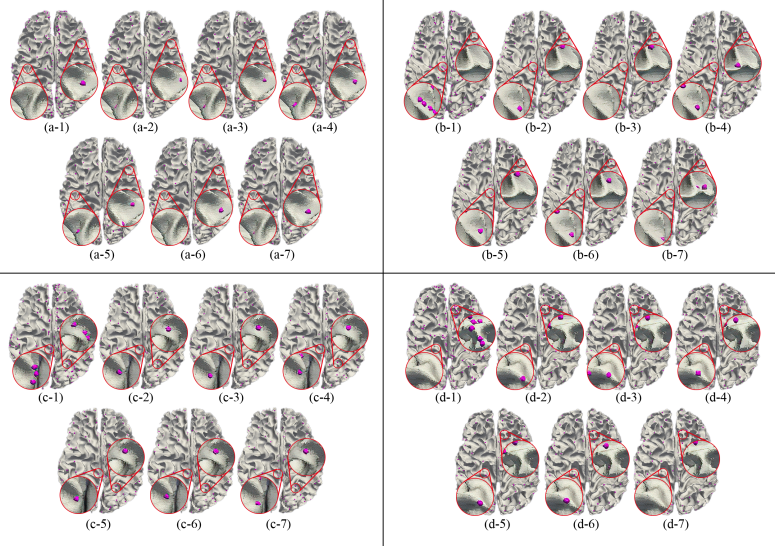


**(A) (B)**

Figure S2: The 3-Hinges regions (A)/centroids (B) visualized results using three features. Letters (a-d) represent different individuals. Numbers (1-7) indicate label, area+curv+sulc, area+sulc+thickness, area+sulc+volume, curv+sulc+thickness, curv+sulc+volume, and sulc+thickness+volume, respectively.


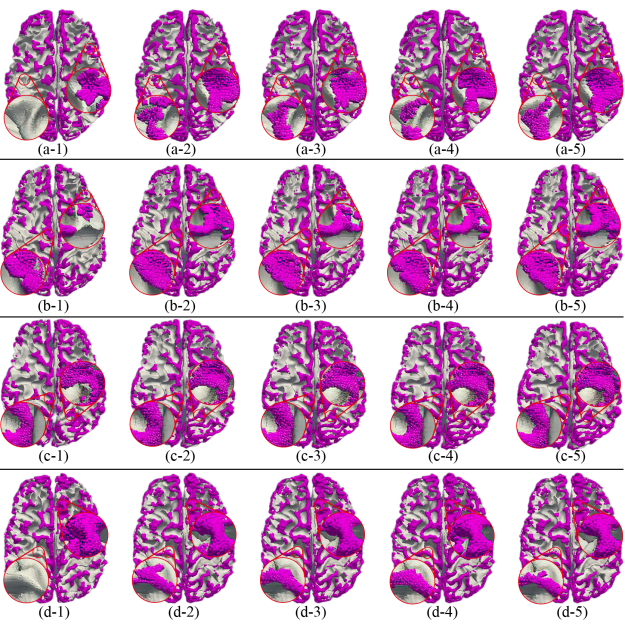

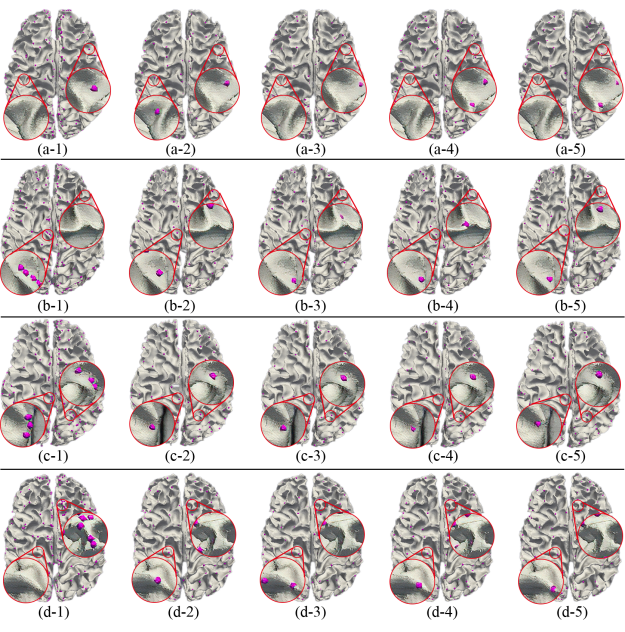


**(A) (B)**

Figure S3: The 3-Hinges regions (A)/centroids (B) visualized results using four features. Letters (a-d) represent different individuals. Numbers (1-5) indicate label, area+curv+sulc+thickness, area+curv+sulc+volume, area+sulc+thickness+volume, and curv+sulc+thickness+volume, respectively.


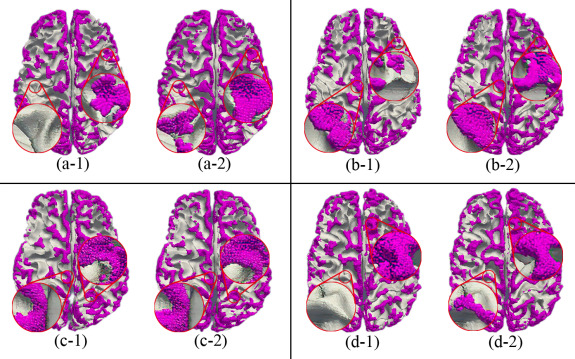

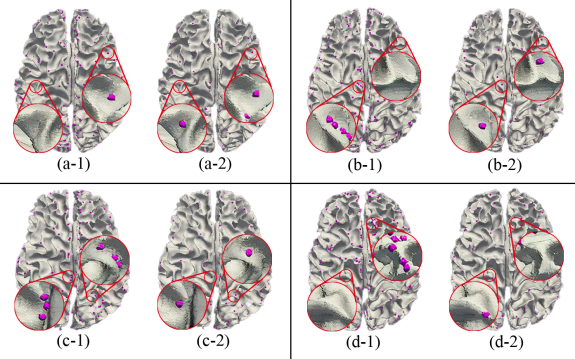


**(A) (B)**

Figure S4: The 3-Hinges regions (A)/centroids (B) visualized results using five features. Letters (a-d) represent different individuals. Numbers (1-2) indicate label, area+curv+sulc+thickness+volume, respectively.
